# Epidemiology and treatment of proximal femoral fractures in the elderly U.S. population

**DOI:** 10.1038/s41598-023-40087-8

**Published:** 2023-08-05

**Authors:** Nike Walter, Dominik Szymski, Steven M. Kurtz, David W. Lowenberg, Volker Alt, Edmund C. Lau, Markus Rupp

**Affiliations:** 1 Department of Trauma Surgery, University Medical Center Regensburg, Franz-Josef-Strauß-Allee 11, 93053 Regensburg, Germany; 2 Implant Research Center, Drexel University, Philadelphia, USA; 3 Department of Orthopaedic Surgery, Stanford University School of Medicine, Stanford, CA USA; 4 Exponent Inc., Menlo Park, USA

**Keywords:** Health care economics, Medical research, Epidemiology

## Abstract

Proximal femoral fractures are a serious complication, especially for elderly patients. Detailed epidemiological analyzes provide a valuable resource for stakeholders in the health care system in order to foresee future development possibly influenceable by adaption of therapeutic procedures and prevention strategies. This work aimed at answering the following research questions: (1) What are the incidence rates of proximal femoral fractures in the elderly U.S. population? (2) What is the preferred treatment procedure for these fractures? Proximal femoral fractures occurred between January 1, 2009 and December 31, 2019 in patients ≥ 65 years were identified from the Medicare Physician Service Records Data Base. The 5% sample of Medicare beneficiaries, equivalent to the records from approximately 2.5 million enrollees formed the basis of this study. Fractures were grouped into head/neck, intertrochanteric, and subtrochanteric fractures. The overall incidence rate, age and sex specific incidence rates as well as incidence rate ratios were calculated. Common Procedural Terminology (CPT) codes were used to identify procedures and operations. In 2019, a total number of 7982 femoral head/neck fractures was recorded. In comparison to 9588 cases in 2009, the incidence substantially decreased by 26.6% from 666.7/100,000 inhabitants to 489.3/100,000 inhabitants (z =  − 5.197, p < 0.001). Also, in intertrochanteric fractures, a significant decline in the incidence by 17.3% was evident over the years from 367.7/100,000 inhabitants in 2009 to 304.0 cases per 100,000 inhabitants in 2019 (z =  − 2.454, p = 0.014). A similar picture was observable for subtrochanteric fractures, which decreased by 29.6% (51.0 cases per 100,000 to 35.9 cases per 100,000) over the time period (z =  − 1.612, p = 0.107). Head/neck fractures were mainly treated with an arthroplasty (n = 36,301, 40.0%). The majority of intertrochanteric fractures and subtrochanteric fractures received treatment with an intramedullary device (n = 34,630, 65.5% and n = 5870, 77.1%, respectively). The analysis indicated that the incidence of all types of proximal femoral neck fractures decreased for the population of elderly patients in the U.S. within the last decade. Treatment of head and neck fractures was mainly conducted through arthroplasty, while intertrochanteric and subtrochanteric fractures predominantly received an intramedullary nailing.

## Introduction

With increasing life expectancy of the general population, the numbers of trauma patients are expected to increase, especially in the elderly^1^. Already in 1997 Gullberg et al. calculated that femoral fractures would double from 1990 to 2025 worldwide and double again by 2050 with a range between 7.3 and 21.3 million fractures worldwide^2^. Other analysis estimated an increase from 1.26 million in 1990 to 4.5 million by 2050^3^. In particular, proximal femoral fractures are an important health-care concern. Besides the morbidity and mortality associated with them, direct costs are enormous with long hospitalization periods and required rehabilitation^4^. The treatment of proximal femoral fractures was ranked the 13th most expensive diagnoses based on Medicare calculations in 2011^5^. Costs for the initial hospitalization and one year health-care were estimated at approximately USD 43,000 per patient^6^.

Detailed epidemiologic evaluation provides a valuable resource for stakeholders in the health care system in order to foresee future development possibly influenceable by adaption of therapeutic procedures and prevention strategies. We have aimed to answer the following questions based on nationwide registry data: (1) What are the overall incidence rate as well as age- and sex adjusted incidence rates of head/neck femoral fractures, intertrochanteric fractures, and subtrochanteric fractures in the elderly U.S. population? (2) What is the preferred treatment procedure for these fractures?

## Methods

Proximal femoral fractures occurring between January 1, 2009 and December 31, 2019 were retrieved from the Medicare Physician Service Records Data Base. These records included services provided by medical offices, clinics, hospitals, emergency departments, skilled nursing facilities, and other healthcare institutions. The Centers for Medicare and Medicaid Services (CMS) compiled these records, which were deidentified and made available for research, known as the Limited Data Set (LDS). These LDS data are based on a 5% sample of Medicare beneficiaries, equivalent to the medical records from approximately 2.5 million enrollees. The 5% LDS records are sampled systematically across the entire Medicare population without varying the sampling fraction by region, age group, or any other factors. Each year, the 5% LDS data captured 18,000 patients with femur fractures and approximately 140,000 femur fracture–related physician records. CMS replaced the beneficiary’s identity with a synthetic and unique ID in the LDS data sets, which allowed patients to be followed longitudinally for survivorship and outcomes analyses. The population of interest included elderly Medicare patients (aged 65 and above).

The International Classification of Diseases, Ninth and Tenth Revisions, were used to identify femoral fractures from these physician records. Diagnoses in claims submitted before October 1, 2015, were recorded in ICD-9-CM and thereinafter in ICD-10-CM. Several steps were implemented to ensure that the identified fracture was true and was a new fracture. First, only records with fracture diagnosis listed as the primary diagnosis were retained. Second, there must be no fracture record of the same type in the previous 1 year. Concurrent fracture of different parts of the femur (e.g., a head/neck fracture) was not uncommon and included. Some patients did experience the same type of fracture more than once during the 10-year study period. But from one fracture to the next, a minimal interval of 1 year was required to ensure that the next fracture was not associated with continued care for the previous fracture. Third, for fractures coded using ICD-10, the 7th digit must be “A”, “B”, or “C”, indicating a new encounter with that condition. Fractures with diagnoses indicating aftercare for healing of fracture or codes that indicated malunion or nonunion would not be counted because these conditions were consistent only with pre-existing fractures^7^.

Surgical treatment following femoral fracture was also investigated. In these physician records, the Common Procedural Terminology (CPT) codes were used to identify procedures and operations. Surgical procedures involving the hip, pelvis, and femur are in the 27,000–27,599 range.

The incidence of femoral fractures was calculated by year and the rate of fracture was estimated by dividing the fracture frequency with the corresponding Medicare enrollments. The overall incidence rate and age and sex specific incidence rates were calculated. Incidence rate ratios (IRR) with the corresponding 95% confidence interval (CI) were calculated by dividing the incidence in 2019 by the incidence of the preceding year. Incidence rates were compared using the two-sample z-test^8^.

Ethical review committee statement: This study is based on data from the Medicare Physician Service Records Data Base. These records encompassed diagnoses and treatments rendered in medical offices, outpatient clinics, hospitals, emergency departments, skilled nursing home, and other healthcare facilities. They were compiled by the Centers for Medicare and Medicaid Services (CMS), and after deidentification were made available for researcher, known as the Limited Data Set (LDS). Since the CMS data is deidentified, IRB approval and informed consent requirements were waived by the ethic committee of the University Hospital Regensburg, Germany. This work was performed in accordance with the Declaration of Helsinki.

### Ethics approval

Since the CMS data is deidentified, IRB approval was waived by the ethic committee of the University Hospital Regensburg, Germany. This work was performed in accordance with the Declaration of Helsinki.

## Results

### Incidence

**Figure 1:**
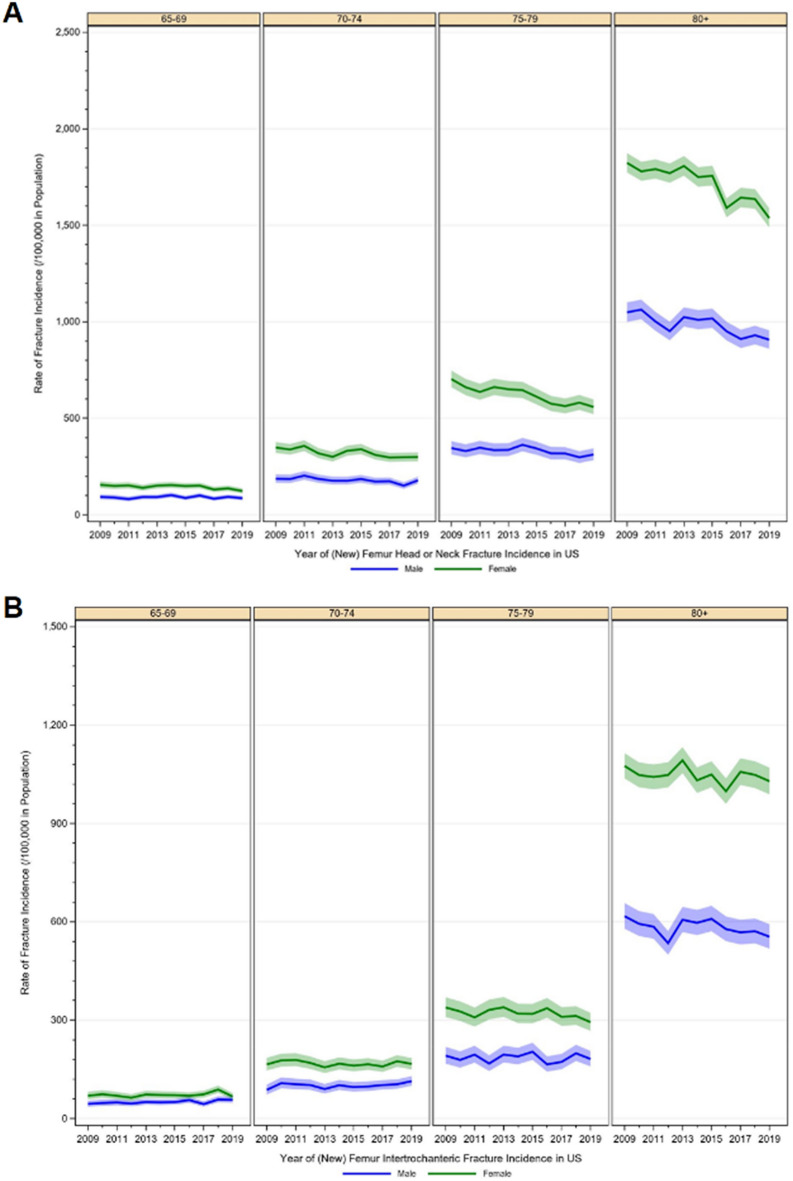

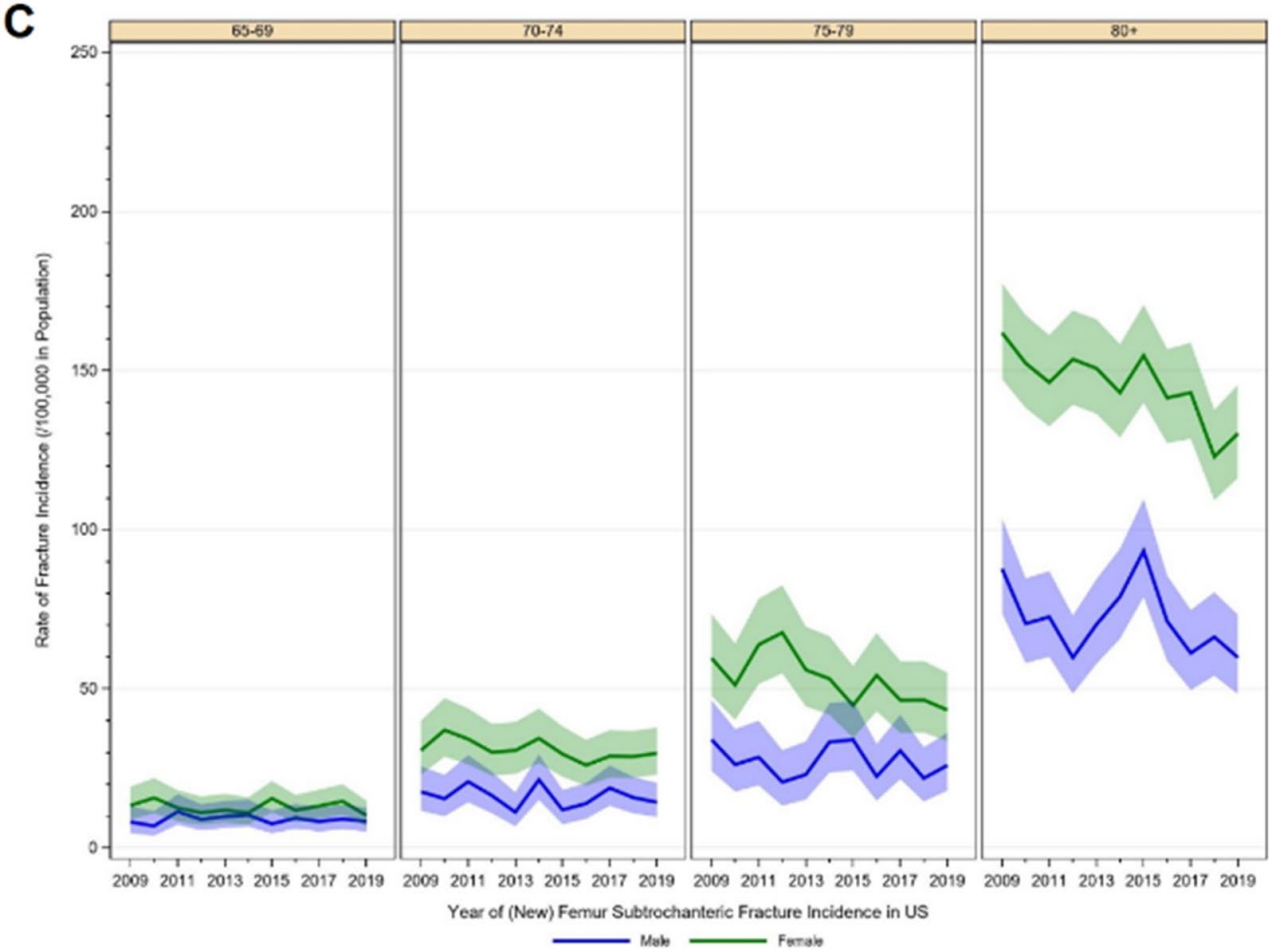
Historic development of age- and sex specific incidence rates of (**A**) femoral head or neck fractures, (**B**) femoral intertrochanteric fractures, (**C**) femoral subtrochanteric fractures.

**Figure 2:**
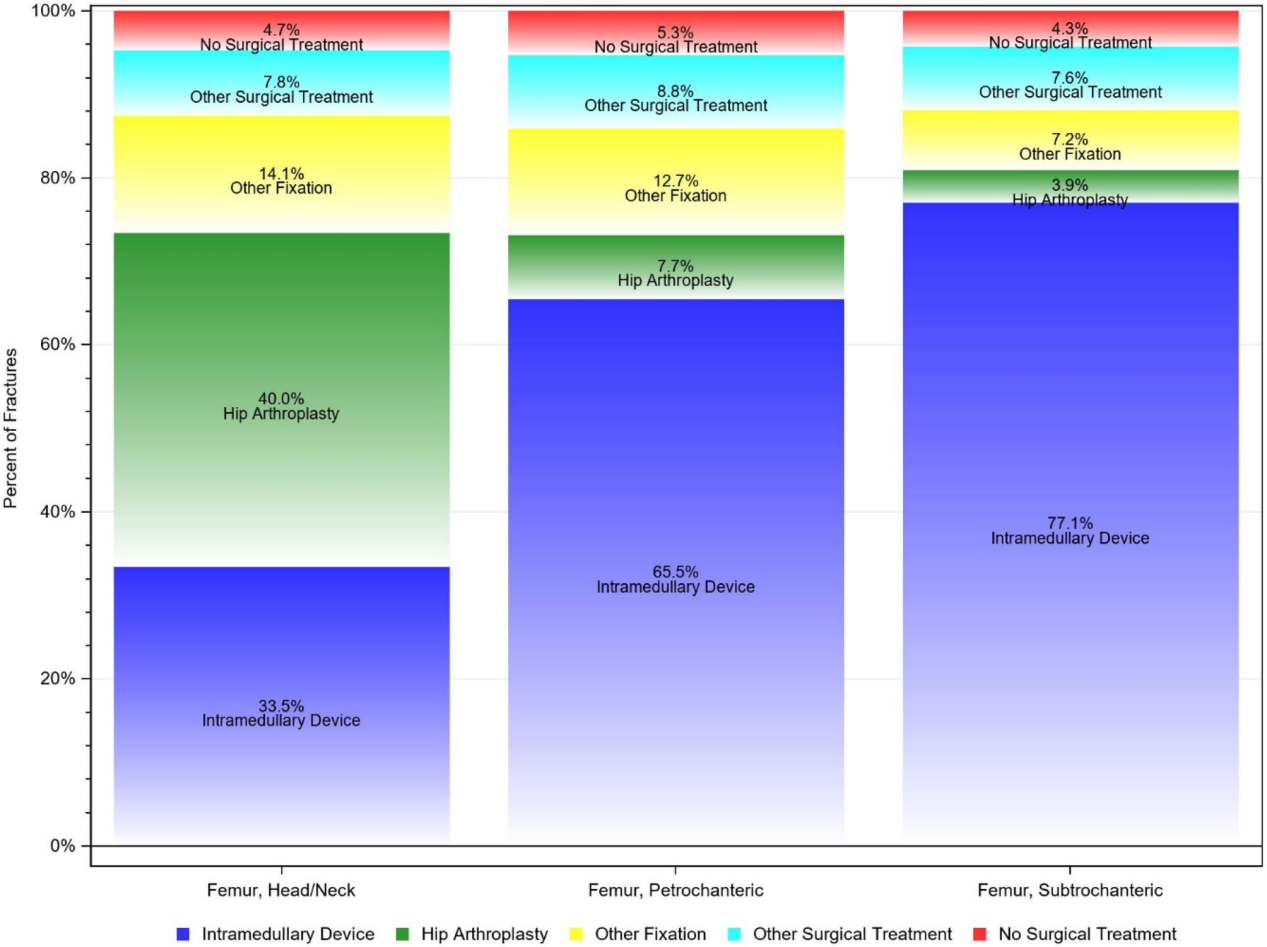
Treatment received within 30 days after fracture.

In 2019, a total number of 7982 femoral head/neck fractures was recorded. In comparison to 9,588 cases in 2009, the incidence substantially decreased by 26.6% from 666.7/100,000 inhabitants to 489.3/100,000 inhabitants (z =  − 5.197, p < 0.001). Also, in intertrochanteric fractures, a significant decline in the incidence by 17.3% was evident over the years from 367.7/100,000 in 2009 to 304.0 cases per 100,000 inhabitants in 2019 (z =  − 2.454, p = 0.014). A similar picture was observable for subtrochanteric fractures, which decreased by 29.6% (51.0 cases per 100,000 to 35.9 cases per 100,000) over the time period (z =  − 1.612, p = 0.107) (Table 1). All three fracture types affected more women than men, and the incidence increased with age (Fig. 1). Head/neck fractures were mainly treated with an arthroplasty (n = 36,301, 40.0%), followed by the use of an intramedullary device (33.5%). The majority of intertrochanteric fractures and subtrochanteric fractures received treatment with an intramedullary device (n = 34,630, 65.5% and n = 5870, 77.1%, respectively) (Fig. 2).

**Table 1:** Historic development of proximal femoral fracture diagnoses between 2009 through 2019.

Year	Head/neck				Intertrochanteric				Subtrochanteric			
	Total	Incidence/100,000	IRR	p-value	Total	Incidence/100,000	IRR	p-value	Total	Incidence/100,000	IRR	p-value
2009	9588	666.7	–	–	5289	367.7	–	–	734	51.0	–	–
2010	9441	647.5	0.97	0.596	5249	360.0	0.98	0.776	658	45.1	0.88	0.558
2011	9405	636.4	0.98	0.757	5188	351.1	0.98	0.738	706	47.8	1.06	0.780
2012	9162	606.9	0.95	0.403	5057	335.0	0.95	0.538	663	43.9	0.92	0.684
2013	9271	605.3	1.00	0.963	5281	344.8	1.03	0.707	633	41.3	0.94	0.778
2014	9103	588.6	0.97	0.629	5071	327.9	0.95	0.514	650	42.0	1.02	0.939
2015	9010	575.6	0.98	0.703	5107	326.3	1.00	0.950	670	42.8	1.02	0.931
2016	8490	529.7	0.92	0.168	5021	313.3	0.96	0.607	642	40.1	0.94	0.767
2017	8337	518.1	0.98	0.720	5055	314.1	1.00	0.974	627	39.0	0.97	0.901
2018	8307	512.8	0.99	0.869	5,151	318.0	1.01	0.877	596	36.8	0.94	0.800
2019	7982	489.3	0.95	0.458	4,959	304.0	0.96	0.575	586	35.9	0.98	0.916

*IRR* incidence rate ratio.

## Discussion

The present analysis provides a comprehensive investigation of age- and sex specific incidence rates of head/neck femoral fractures, intertrochanteric fractures, and subtrochanteric fracture, as well as treatment procedures in elderly patients in the US.

In this study, a decrease in the incidence of proximal femoral fractures in the elderly U.S. population could be observed from 2009 through 2019. Femoral head/neck fractures decreased by 26.6%, intertrochanteric fractures by 17.3%, and subtrochanteric fractures by 29.6% in this patient population subset. In line with these findings, Brauer et al. examined trends in femoral fracture incidence based on Medicare data from 1985 to 2005. The authors reported a decline of 24.5% in women and 19.2% in men, which corresponded to an increased use of bisphosphonates and thus, might be attributable to advances in the diagnosis and treatment of osteoporosis^9^. Another population-based cohort study including 10,552 individuals revealed an age-adjusted decrease of 4.4% (95% CI 6.8–1.9%) per year from 1970 to 2010. This was attributed to a decrease in smoking and heavy drinking and not solely with improvement in treatment^10^. Other analyses showed that femoral fracture rates declined between 2002 and 2005, but have increased and plateaued in 2013^11^. Also, in Canada a decline in incidence of these fractures by 32% in females and by 25% in males was observed between 1985 and 2005^12^. In contrast, a comparable analysis between 2009 and 2019 based on a nationwide registry data of Germany reported that femoral neck and intertrochanteric fractures were the most frequent ones, whereby incidences increased by 23% and 24%, respectively, and subtrochanteric fractures increased by 30%^13^. Methodological and data variations may account for these differences. While in this analysis, the targeted population only included patients aged 65 years or older, the study conducted in Germany, found an increase in the incidence of femoral neck fractures between 2009 and 2019 already in the age group of 50 to 59 years. Also, while highest growth rates were described in the population aged 90 and older with 68.9%, the lowest with 16.9% was found in the subpopulation between 70 and 79 years old^14^. In Italy, Japan and the Netherlands increasing fracture rates were reported recently which is contrary to the decreasing incidence in the U.S. and Canada^15–17^. The disparity in incidence rates between the U.S and other countries also suggests that there may be contributing factors beyond risk factors alone that have influenced these differing trends. This may include variations in healthcare systems and differences in access to preventive measures, and early diagnosis. In addition, demographic differences and the aging population structure could contribute to the observed variations. Further, lifestyle factors, such as physical activity levels and nutrition, might differ between the U.S. and other countries, which can influence bone health and fracture risk. As risk factors the age over 65, female sex, reduced bone mineral density, alcohol or tobacco abuse, chronic diseases, reduced body mass index and reduced activity level were mentioned in the literature^3,10,18,19^. A possible explanation of the incidence reduction in our analysis is the lower rate of alcohol and tobacco use in the U.S.^10^. In addition, comprehensive and targeted initiatives to reduce falls in the elderly population were implemented in the U.S. potentially contributing to the decline in incidence rates^20,21^. Concurrent in Finland Kannus et al. found a reduction in the incidence of proximal femoral fractures and mentioned improved osteoporosis diagnostics and therapy, fall prevention programs, changed eating habits and increased physical functionality in elderly peoples as main reasons^22^.

Decision-making on appropriate treatment of proximal femoral fractures is dependent on fracture localization, age and demand of the patient and preexisting joint degeneration^19,23^. Basically, a distinction with all advantages and disadvantages must be made between arthroplasty and osteosynthesis as treatment of proximal femoral fractures. While arthroplasties are associated with a higher blood loss and rate of infection, therapy through osteosynthesis demonstrated the risk of nonunion, necrosis and joint degeneration^19,23–25^. In our analysis of Medicare data the highest rate of joint replacement was reported for head and neck fractures. In Germany similar pattern with arthroplasty implantation rates of up to 79.4% was detected for intracapsular neck fractures^14^. In line, Saul et al. mentioned in neck fractures high treatment numbers performed arthroplasties and sliding hip screws^26^. However, to note, it has also been shown that patients with femoral neck fractures treated with intramedullary nailing had a shorter time in bed and better functional recovery, lower incidence of complications and higher long-term joint function with better quality of life compared to patients treated with plate fixation^27^. Further, a recent meta-analysis of ten studies including 991 patients concluded that a reconstruction nail is a more efficient and safer treatment than a hollow screw and plate for patients with femoral shaft and femoral neck fracture^28^.

Intertrochanteric and subtrochanteric fractures were mainly treated with proximal femoral nailing which is in line with previously reported results^26^. In particular for intertrochanteric and subtrochanteric femoral fractures intramedullary devices are deemed the best biomechanical option through intramedullary buttress and distal locking^29^. Sliding hip screws are also an option and demonstrated equal results with poorer pain reduction and mobilization scores compared to intramedullary nailing^30^. In line with our findings concerning the use of intramedullary devices, also other authors have reported the changing pattern of practice, whereby the intramedullary nail fixation rate for intertrochanteric fractures was shown to be increased from 3% in 1999 to 67% in 2006^31^. Hip replacement surgeries provide also an alternative in intertrochanteric and subtrochanteric fractures, but remain technically challenging and are not classified as first-line-therapy^29^.

### Limitations

To begin with, the analysis is based on a 5% sample of Medicare beneficiaries, which equates to records from about 2.5 million enrollees aged over 65 years. The 5% LDS records are sampled systematically across the entire Medicare population without varying the sampling fraction by region, age group, or any other factors. Thus, the 5% sample is a true and representative cross-section of the Medicare population. Additionally, because the sampling weight is a constant, including weight would not affect any of the results. However, since this represents only a small fraction of the overall U.S. population, the generalization of the results is limited and the incidence may be underestimated. The study relies on Medicare data, which is essentially a collection of administrative claims records. This type of data has limitations for orthopedic outcomes research^32,33^. Further, the analysis relies on the accuracy of coding and data management, which can only be verified through Medicare’s claim processing system. Moreover, clinical measures such as radiologic imaging findings and other indicators like blood chemistry and organ function tests are not captured by the diagnosis or procedure codes in the data. While an individual record may contain errors, systematic errors are unlikely to be present throughout millions of records in the entire Medicare system. This is due to the broad range of facilities submitting these claims and the hundreds of millions of records processed by Medicare, which is a unique strength of the system.

## Conclusion

The analysis indicated that that femoral neck, intertrochanteric and subtrochanteric fractures incidence decreased for the population of patients aged 65 and older in the U.S. between 2009 and 2019. Treatment of head and neck fractures was mainly performed through arthroplasty, while intertrochanteric and subtrochanteric fractures predominantly received an intramedullary nailing. Despite decreasing incidences, proximal femoral fractures represent a clinical challenge in geriatric trauma care.

## Data Availability

The datasets used and/or analysed during the current study available from the corresponding author on reasonable request.
